# Lower levels of plasma syndecan-4 are associated with loss of body weight and fat-free mass after bariatric surgery

**DOI:** 10.1186/s13104-024-06822-8

**Published:** 2024-06-15

**Authors:** Catia Martins, Marthe Isaksen Aukan, Maria De Luca

**Affiliations:** 1 Department of Nutrition Sciences, University of Alabama at Birmingham (UAB), Birmingham, AL USA; 2 Obesity Research Group, Department of Clinical and Molecular Medicine, Faculty of Medicine, Norwegian University of Science and Technology (NTNU), Trondheim, Norway; 3 Centre of Obesity and Innovation (ObeCe), Clinic of Surgery, St. Olav University Hospital, Trondheim, Norway

**Keywords:** Extracellular matrix, Heparan sulfate proteoglycans, Severe obesity, Skeletal muscle, Bariatric procedure

## Abstract

**Objective:**

Bariatric surgery induces a significant loss of both fat mass (FM) and fat-free mass (FFM). The proteoglycan receptor syndecan-4 (SDC4) plays a crucial role in adipose tissue and skeletal muscle functions. Thus, this study was performed (i) to assess plasma SDC4 levels after both Sleeve Gastrectomy (SG) and Roux-en-Y Gastric Bypass (RYGB) surgeries, and (ii) to explore potential associations with changes in body composition variables.

**Results:**

Twenty-six patients (17 females) with severe obesity underwent SG (*n* = 13) or RYGB (*n* = 13) and were followed up to 1 year (1Y). Body weight, FM, FFM, and SCD4 were measured at baseline (BL), and at week 11 (W11) and 1Y after surgery. Independently of procedure, there was a significant body weight loss at W11, with an average FM and FFM reduction of 13.7 ± 0.6 kg and 5.3 ± 0.5 kg, respectively. Participants continued to lose weight afterwards, with a total weigth loss of 38.2 ± 1.5 kg at 1Y. No associations were found at BL between SDC4 levels and any anthropometric variable; however, SDC4 levels were lower than BL at both W11 and 1Y, independently of type of surgery. Additionally, changes in SDC4 between BL and 1Y were positively correlated with weight and FFM loss during the same period.

**Trial registration:**

ClinicalTrials.gov NCT04051190 on 09/08/2019.

## Introduction

Bariatric surgery procedures, such as SG and RYGB, are the most effective long-term treatments for severe obesity, inducing large and sustained FM loss, along with improvements in overall and metabolic health [1, 2]. However, a downside of bariatric surgery is a variable loss of FFM occurring within 1Y post-surgery, which is independent of sex, age, or surgical technique [3]. Loss of skeletal muscle integrity and bone strength can have detrimental consequences on the patients’ functional capacities and impact their ability to conduct daily life activities [4]. Thus, research efforts directed towards a better understanding of the factors contributing to bariatric surgery-related loss of FFM are of paramount importance.

SDC4 is a member of the SDC family of heparan sulfate proteoglycans, which consists of four transmembrane proteins (SDC1 to 4). While the other SDCs present a tissue-specific expression pattern, SDC4 has a widespread distribution [5]. All of them are, however, characterized by an extracellular domain (ectodomain) with attachment sites for glycosaminoglycan chains (e.g. heparan sulfate and chondroitin sulfate) that mediate interactions with a wide array of ligands, like ECM components, soluble growth factors, morphogens, and cytokines [6]. The extracellular domain of the SDCs is followed by a highly conserved transmembrane domain and a short cytoplasmic tail. The latter contains sites for cytoskeletal proteins and protein kinases that allow members of the SDC family to control cell behavior [7, 8]. As a result, SDCs are involved in several biological mechanisms, including tissue repair and regeneration [9] and inflammation [10].

Recent evidence strongly suggests that SCD4 plays a pivotal role in the control of body composition by regulating processes related to fat storage, muscle development, and bone fracture repair [11–15]. For instance, genetic studies in mice harboring a *Sdc4* global knockout allele demonstrated that SDC4 deficiency promotes diet and sex-specific changes in percentage of body fat and lean mass and metabolic outcomes [12, 16, 17]. Furthermore, SDC4 is a mechanosensor [18–24] and alterations in mechanical properties of adipose tissue have been shown to occur following bariatric surgery [25]. The ectodomain of SDC4 is constitutively released from the cell surface through a process mediated by matrix metalloproteinases (MMP) that can be accelerated by various physiological agents [26] and mechanical stress [22, 27]. Thus, based on the above observations, the objective of the present study was to assess whether circulating SDC4 levels might correlate with bariatric surgery-induced changes in body composition.

## Main text

### Methods

#### Study design and participants

Our investigation was performed in 26 patients with severe obesity (BMI: 41 ± 4 kg/m^2^; age 44 ± 12 years) who underwent either SG (*n* = 13) or RYGB (*n* = 13). These patients were previously enrolled to participate in a non-randomized controlled trial specifically designed to assess how a similar weight loss induced by very-low energy diet (VLED) alone, or VLED in combination with SG or RYGB, impacts different domains of appetite regulation [28]. Due to the objective of this study, only participants in the bariatric groups were included in the present analysis.

Details on the study design were previously reported [28]. Briefly, adults with severe obesity scheduled for SG or RYGB at two local hospitals in the Central Norway Health Region were recruited between September 2019 and January 2022. Participants had to be weight stable (self-reported) (< 2 kg body weight change over the last three months) and not enrolled in any other obesity treatment or behavioral program. Exclusion criteria included patients who had previously undergone bariatric surgery, were using medication known to affect metabolism or appetite, had a current cancer diagnosis, substance abuse, as well as present a psychiatric diagnose that precluded bariatric surgery (such as eating disorders). Patients who were scheduled for bariatric surgery initiated the VLED two weeks prior to surgery and continued for another eight weeks afterwards. They were then instructed to consume only fluid food packs the first weeks post-operatively, gradually increasing the texture of the food. All 26 participants who underwent bariatric surgery completed BL and W11 assessments, but only 21 came back for a follow up 1Y after surgery.

#### Outcome variables

After an overnight fast (at least 10 h), participants came to the Obesity outpatient clinic at St. Olav’s University Hospital before start of the dietary intervention (BL), after 10 weeks (W11), and after 1Y to measure body weight and composition and collect a blood sample. Body weight, FM, and FFM were measured using Air-displacement plethysmography (BodPod, COSMED, Rome, Italy). The proportion of FFM loss from total weight loss was calculated as reported by Nuijten and colleagues [3].

Blood samples were collected in a 4 ml EDTA-coated tube, centrifugated immediately and kept at -80 C until analysis. Plasma SDC4 levels were assessed blinded to the clinical data using a high-sensitivity enzyme-linked immunosorbent assay kit (Immuno-Biological Laboratories, Minneapolis, MN, USA), following the manufacturer’s instructions. Standards were within the limits of detection and samples’ intra-assay and inter-assay coefficients of variation were < 7% and < 3%, respectively.

#### Statistical analysis

The statistical analysis was run using SPSS, version 27 (SPSS Inc., Chicago, IL) and SAS 9.4 (SAS Institute Inc, Cary, NC, USA). Residuals were checked for normality using the Shapiro-Wilk test and visual inspection of QQ plots and histograms. A linear mixed model with fixed effect for time (BL, 11 W, and 1Y), group (SG, and RYGB), and their interaction was used to describe the changes in anthropometrics and SDC4 over time. A Tukey-Kramer *post-hoc* test was used to assess significant comparisons. Correlation and multiple linear regression analyses were used to explore the associations between SDC4 alterations and changes in body weight and composition. *P*-values < 0.05 were considered statistically significant.

## Results

Table 1 shows the mean characteristics of the SG and RYGB groups over time. There was a main effect of time for all anthropometric variables. However, no differences in any anthropometric variables between groups at any time point, or in changes over time were observed. Participants lost on average 19.1 ± 0.7 kg (16%) at W11, FM decreased by 13.7 ± 0.6 kg, and FFM by 5.3 ± 0.5 kg, with no differences between groups. Participants continued to lose weight between W11 and 1Y, resulting in a total weight loss at 1Y of 38.2 ± 1.5 kg, independently of surgical procedure. The proportions of FFM loss from total weight loss at W11 and 1Y were 27% and 30%, respectively.

**Table 1:** Study variables over time

	Baseline			Week 11		1 Year		*p-value*			
	SG	RYGB		SG	RYGB	SG	RYGB	*Time*	*Group*		Time* group
*N*	13	13		13	13	9	12				
Age	38.9 ± 2.6	48.5 ± 3.3							NS		
Females %	69	62							NS		
Body weight (kg)	118.9 ± 4.1	120.7 ± 4.5		99.8 ± 3.4	101.7 ± 3.5	82.4 ± 5.8	82.1 ± 4.5	< 0.001	0.832		0.764
BMI (kg/m^2^)	40.1 ± 0.9	41.8 ± 1.4		33.7 ± 0.8	35.1 ± 1.5	27.5 ± 1.2	28.4 ± 1.8	< 0.001	0.405		0.996
Weight Δ (kg) from BL				-20.5 ± 1.3	-19.1 ± 1.2	-39.4 ± 1.8	-37.5 ± 2.7				
Weight Δ (%) from BL				− 17.2	− 15.8%	− 32.8%	− 31.7%				
FM (%)	47.7 ± 1.2	47.5 ± 2.0		42.5 ± 2.1	44.1 ± 2.3	28.1 ± 2.8	29.1 ± 3.5	< 0.001	0.742		0.838
FM (kg)	56.7 ± 2.3	57.2 ± 3.3		42.5 ± 2.1	44.1 ± 3.3	23.5 ± 3.2	24.5 ± 4.0	< 0.001	0.672		0.728
FFM (kg)	62.2 ± 2.6	63.2 ± 2.8		57.3 ± 2.3	57.5 ± 2.5	58.9 4.0	57.4 ± 3.1	< 0.001	0.864		0.614
SDC4 (pg/mL)	1759 ± 175	1844 ± 175		1385 ± 198	1527 ± 198	1159 ± 210	1642 ± 187	0.011	0.282		0.407

Data presented as estimated marginal means ± SEM. BMI: body mass index; FM: fat mass; FFM: fat free mass; SG: Sleeve Gastrectomy; RYGB: Roux-en-Y Gastric Bypass; Δ: change; NS: not significant

**Fig. 1:**
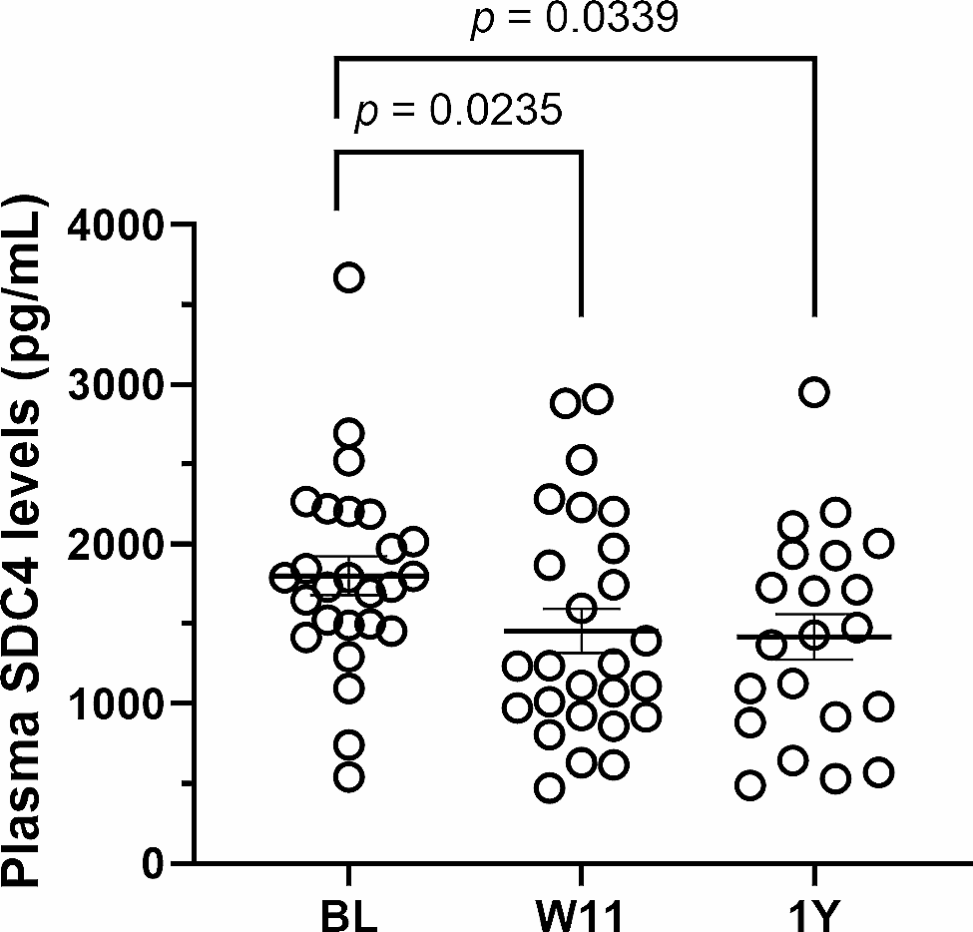
Plasma levels of shed SDC4 before and after gastric bypass. BL: baseline; W11: week-11; 1Y: 1-year

A significant main effect of time was also observed for SDC4; however, there was no effect of surgical group or time-by-group interaction (Table 1). Shed SDC4 levels in postoperative patients decreased over time independently of bariatric surgery technique, with values being significantly lower than BL at both W11 (19%) and 1Y (22%) (Fig. 1).

**Fig. 2:**
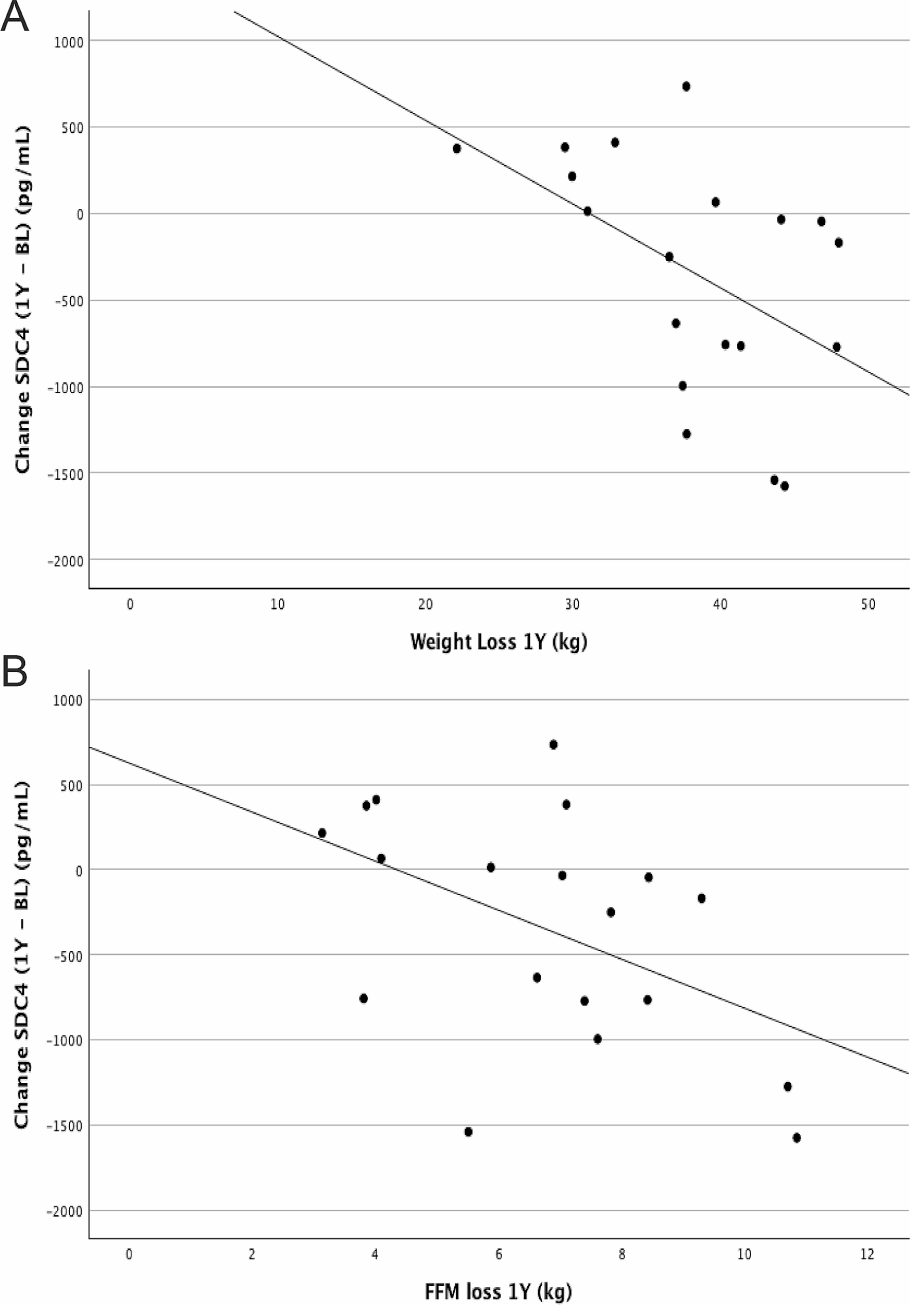
Scatterplots for the association of shed SDC4 changes between baseline (BL) and 1 year (1Y) with weight (panel A) and FFM (panel B) loss during the same period

Plasma SDC4 levels at BL were not associated with age, BMI, weight, FM or FFM, but changes in SDC4 from BL to 1Y were positively correlated with loss of body weight (*r* = 0.577, *p* = 0.012, *n* = 18) (Fig. 2A) and FFM (*r* = 0.52, *p* = 0.020, *n* = 18) (Fig. 2B) during the same period. Moreover, as shown in Table 2, weight loss at 1Y was a significant predictor of SDC4 plasma concentrations at 1Y, after adjusting for shed SDC4 at BL and bariatric surgery group.

**Table 2:** Multivariate linear regression model predicting SDC4 plasma concentrations at 1 year after bariatric surgery

Predictor	ß-coefficient (95% CI)	*p*-value	Adjusted R^2^
Multivariate model		0.004	0.330
Constant	822 (-59.7, 1704)	0.066	
SDC4 at BL (pg/mL)	-0.43 (-0.78, -0.09)	0.017	
Group	315 (-80.7, 710)	0.114	
Weight loss at 1Y (kg)	32.5 (11.2, 53.8)	0.004	

Group: Sleeve gastrectomy and Roux-en-Y gastric bypass. Each unstandardized ß-coefficient represents 1 pg/mL per unit of the predictor. Variance inflation factors (VIF) <2.2

## Discussion

It is well-documented that, apart from significantly reducing body fat, bariatric surgery leads to a variable amount of FFM reduction within 1Y post-surgery [3]. Excessive loss of FFM (> 25% of weight loss coming from FFM) occurs in approximately 14–46% of bariatric surgery patients [29]. Accordingly, the present study showed that patients with severe obesity not only lost FM, but also significant amount of FFM after 10 weeks of surgery. The proportion of FFM loss with respect to weigh loss was on average 30% at 1Y, independently of the bariatric procedure. Several studies have reported a significant reduction (ranging from 24 to 89%) in long-term mortality rates for bariatric surgery patients, when compared with non-bariatric surgical controls with severe obesity [30]. This reduction is partially due to improvement in comorbidities associated to weight loss [30]. Yet, the long-term excessive loss of FFM after bariatric surgery can have harmful effects on the patients’ daily living activities, making interventions to mitigate such loss vital [3]. Understanding the mechanisms of action for bariatric surgery-related loss of FFM will likely help in identifying biomarkers for FFM loss that could inform effective intervention guidlines.

ECM remodeling is essential for adipose tissue growth and expansion [31]. Meen and colleagues [32] recently reported that obesity is associated with distorted expression of proteoglycans and other ECM-related proteins in adipose tissue and that adipose tissue repair is still observed 1Y after bariatric surgery. These authors also reported an increase in *SDC4* and a decrease in *MMP-9* mRNA expression after bariatric surgery (SG and RYGB) in both subcutaneous and visceral adipose tissue [32]. Given that MMP-9 is a sheddase of SDC4 [26], these findings suggest that circulating levels of shed SDC4 might be reduced after bariatric surgery. In this regard, the major finding of this study was that gastric bypass-induced loss of body weight was associated with a reduction in circulating SDC4 levels, which was independent of SG or RYGB procedure. In addition, a dose response association was observed at 1Y after gastric bypass, with a greater reduction in plasma SDC4 levels being associated to more weight loss. However, contrary to predictions, changes in plasma SDC4 levels following gastric bypass were not correlated with FM loss, but with FFM loss at 1Y after surgery. A crosstalk exists between white adipose tissue and muscle that is mediated by adipose tissues-secreted signaling mediators, including extracellular vesicles (EV), and is essential to maintain metabolic health [33]. Along with syntenin-1 and ALIX, SDC4 has been identified as an important regulator of exosome biogenesis [34] and exosomes of adipose tissue-derived mesenchymal stem cells can promote skeletal muscle regeneration [35]. Once released from the cell surface, the SDC ectodomains may act as paracrine or autocrine effectors, or compete with cell surface receptors for the same ligand [36]. Thus, a potential explanation for our findings is that the shed SDC4 released during the adipose tissue remodeling occurring in postopeartive patients might affect exosome production of adipose tissue-derived mesenchymal stem cells and ultimately muscle regeneration. An alternative hypothesis is that changes in expression and shedding of SDC4 might take place directly in the skeletal muscle after bariatric surgery. This idea is supported by previous research showing that weight loss after RYGB results in skeletal muscle methylation changes in genes related to cell-matrix adhesion [37]. Due to the nature of our study design, we cannot make causal conclusions. Nevertheless, our results and the above observations strongly motivate further research to better understand whether SDC4 plays a role in the mechanisms responsible for bariatric surgery-related loss of FFM.

It has been reported that weight loss interventions, like bariatric surgery, should be accompanied by a supervised resistance and aerobic exercise program to preserve loss of FFM [38]. However, the beneficial effect of exercise on weight-related outcomes depends on several factors, such as the optimal time, length, intensity, and type of exercise program [39]. As such, the identification of blood-based biomarkers that can predict how patients who have lost a substantial amount of FFM after bariatric surgery respond to a particular exercise program could be valuable. Acute exercise, consisting of 45-minute cycling at a workload equivalent to 70% of the participants’ individual maximum oxygen uptake, has been reported to enhance circulating SDC4 concentrations and skeletal muscle mRNA levels of SDC4 and its sheddases in sedentary men [40]. The results of our study suggest that circulating levels of SDC4 could discriminate between patients who lost excessive FFM within 1Y post-surgery and those who did not. If this is confirmed in follow-up studies, an acute exercise-induced increase in SDC4 levels might play an important role in preserving FFM in the first year after bariatric surgery.

### Limitations

This study has two major strengths. First, weight loss, diet composition, and ketosis level were similar across groups, allowing for the identification of the impact of SG and RYGB alone on the outcome variables. Second, sex distribution, age, baseline anthropometric variables and physical activity levels (not shown) were similar across groups and, therefore, unlikely to have affected the variables of interest. However, this study also suffers from some limitations. First, with this study design, a cause-effect relationship cannot be established. Second, the study is likely underpowered to detect differences between groups. To this end, it cannot be excluded that the lack of association between changes in plasma SDC4 levels and FM loss might be due to the small sample size of the present study. Third, although our findings suggest that circulating SDC4 may be a potential biomarker of FFM loss in patients with severe obesity undergoing bariatric surgery, studies with larger sample sizes are needed to confirm our results.

## Data Availability

The data that support the findings of this study are available upon reasonable request to CM and MD.

## Abbreviations

FM: Fat mass
FFM: Fat-free mass
SDC4: Syndecan-4
SG: Sleeve Gastrectomy
RYGB: Roux-en-Y Gastric Bypass
BL: Baseline
W11: Week 11
1Y: 1 year
MMP: Matrix metalloproteinases
VLED: Very-low energy diet
